# Interferon gamma immunoPET imaging to evaluate response to immune checkpoint inhibitors

**DOI:** 10.3389/fonc.2023.1285117

**Published:** 2023-12-07

**Authors:** Justin B. Hackett, Nicholas Ramos, Stephen Barr, Madeline Bross, Nerissa T. Viola, Heather M. Gibson

**Affiliations:** Karmanos Cancer Institute, Department of Oncology, Wayne State University School of Medicine, Detroit, MI, United States

**Keywords:** interferon gamma, immunoPET imaging, immunotherapy, immune checkpoint inhibitors, radioactive tracer

## Abstract

**Introduction:**

We previously developed a ^89^Zr-labeled antibody-based immuno-positron emission tomography (immunoPET) tracer targeting interferon gamma (IFNγ), a cytokine produced predominantly by activated T and natural killer (NK) cells during pathogen clearance, anti-tumor immunity, and various inflammatory and autoimmune conditions. The current study investigated [^89^Zr]Zr-DFO-anti-IFNγ PET as a method to monitor response to immune checkpoint inhibitors (ICIs).

**Methods:**

BALB/c mice bearing CT26 colorectal tumors were treated with combined ICI (anti-cytotoxic T-lymphocyte-associated protein 4 (CTLA-4) and anti-programmed death 1 (PD-1)). The [^89^Zr]Zr-DFO-anti-IFNγ PET tracer, generated with antibody clone AN18, was administered on the day of the second ICI treatment, with PET imaging 72 hours later. Tumor mRNA was analyzed by quantitative reverse-transcribed PCR (qRT-PCR).

**Results:**

We detected significantly higher intratumoral localization of [^89^Zr]Zr-DFO-anti-IFNγ in ICI-treated mice compared to untreated controls, while uptake of an isotype control tracer remained similar between treated and untreated mice. Interestingly, [^89^Zr]Zr-DFO-anti-IFNγ uptake was also elevated relative to the isotype control in untreated mice, suggesting that the IFNγ-specific tracer might be able to detect underlying immune activity *in situ* in this immunogenic model. In an efficacy experiment, a significant inverse correlation between tracer uptake and tumor burden was also observed. Because antibodies to cytokines often exhibit neutralizing effects which might alter cellular communication within the tumor microenvironment, we also evaluated the impact of AN18 on downstream IFNγ signaling and ICI outcomes. Tumor transcript analysis using interferon regulatory factor 1 (IRF1) expression as a readout of IFNγ signaling suggested there may be a marginal disruption of this pathway. However, compared to a 250 µg dose known to neutralize IFNγ, which diminished ICI efficacy, a tracer-equivalent 50 µg dose did not reduce ICI response rates.

**Discussion:**

These results support the use of IFNγ PET as a method to monitor immune activity *in situ* after ICI, which may also extend to additional T cell-activating immunotherapies.

## Introduction

Modern cancer immunotherapy approaches have improved clinical outcomes for a subset of malignancies, and immune checkpoint inhibitors (ICI) are among the most promising of these modalities. Immune checkpoints are ligand:receptor interactions that regulate the activity of inflammatory cells. These checkpoints are critical for tempering immune activity; they play a major role in slowing the immune response to prevent excessive tissue damage and/or autoimmunity after a trigger, such as an infection (1–3). In cancer, immune checkpoints are coopted by malignant cells to escape immune-mediated attack. FDA-approved ICI therapies have primarily targeted two inhibitory signaling axes: cytotoxic T-lymphocyte-associated protein 4 (CTLA-4) and programmed death 1 (PD-1) with its primary ligand PD-L1. Both CTLA-4 and PD-1/PD-L1 signaling down-regulate the inflammatory effector functions of multiple immune cell subtypes, including CD8^+^ T-cells [reviewed in (4)]. Recently, an antibody targeting lymphocyte-activation gene 3 (LAG-3) received Food and Drug Administration (FDA) approval (5) and several additional targets are undergoing clinical testing (6).

Combination of ICI therapies has proven beneficial for advanced melanoma, lung, and renal carcinomas (5, 7–13). Melanoma is recognized as the most responsive cancer to this therapy. Despite its notable clinical success, durable response to combined ICI in metastatic melanoma is less than 50%, and it remains unclear what factors differentiate which patients will benefit (5, 11–13). PD-L1 expression is an imperfect biomarker that neither precludes nor guarantees response to PD-1/PD-L1-directed therapies (reviewed in (14). Tumor mutational burden is somewhat reliable as a biomarker, with the most highly mutated tumors trending toward improved ICI response rates, but the correlation is not robust (15, 16). Additionally, collecting and analyzing patient samples to evaluate tumor mutational burden can be invasive, expensive, and is not always feasible.

The development of methods to measure immune activity during ongoing therapy also remains a significant challenge. Peripheral immune analyses are currently non-standardized, typically require either knowledge of the antigen(s) of interest or access to adequate tumor tissue to provide said antigen(s), and the results of these assays may not accurately reflect the immune status within the tumor microenvironment (TME). Traditional imaging technologies, such as positron emission tomography (PET) and computed tomography (CT), are utilized to measure tumor volume, but the tumor volume alone may be deceiving if the immunotherapy is driving an influx of beneficial tumor-reactive immune cells. This phenomenon, known as pseudoprogression, mimics tumor growth, but is typically followed by at least a partial response to therapy (17). Given the need for better monitoring options, there has been considerable activity in the development of more relevant immune imaging technologies. The most common PET imaging modality for oncology uses an ^18^F-radiolabeled glucose analog (FDG), which detects metabolically active tumors. Since active immune cells will also take up FDG, it is difficult to distinguish tumor versus immune cell FDG uptake after immunotherapy (18). A small study evaluated whether FDG PET imaging could provide an early prediction of therapeutic response 12-18 weeks after initiation of ICI (19). The authors observed a “flare” defined as >100% standardized uptake value (SUV) increase from baseline without a corresponding increase in tumor volume in 2/7 patients with subsequent complete tumor regression, but an absence of this flare was not predictive of outcomes. More recently, efforts have turned to the development of novel imaging agents designed to specifically measure immune cell presence and/or activity within the tumor, including tracers targeting CD3, CD4, CD8, IL-12, IL-2R, granzyme B, inducible co-stimulator (ICOS), and the immunoPET tracer we have developed against interferon gamma (IFNγ) (20–26).

Initially discovered as an anti-viral cytokine, IFNγ also plays a critical role in the interaction between cancer and the immune system (27, 28). IFNγ is secreted primarily by innate NK and NKT cells, and adaptive Th1-skewed CD4 and cytotoxic CD8 T cells, and IFNγ is a necessary component of an efficacious anti-tumor immune response (29). The clinical importance of IFNγ as an inflammatory mediator is evident; an anti-IFNγ antibody (Emapalumab) has been FDA approved for treating hemophagocytic lymphohistiocytosis, which is driven by IFNγ signaling (30). We have developed an immunoPET tracer utilizing a radiolabeled antibody to IFNγ to monitor response to cancer immunotherapy, and we previously demonstrated that anti-IFNγ PET can identify HER2 cancer vaccine-induced intratumoral immune activation (26). In the current study, we further validated the utility of our IFNγ immunoPET tracer to detect anti-tumor immunity *in situ* using a pre-clinical model that is responsive to ICI. We then evaluated the downstream effect of the tracer antibody on IFNγ signaling by measuring expression of interferon regulatory factor 1 (IRF1) (31). Finally, we tested whether the tracer dose of anti-IFNγ antibody inhibits ICI efficacy.

## Materials and methods

### Mice and cell lines

BALB/c-syngeneic CT26 colorectal cancer cells, a tumor that is characterized as having a high mutational burden without microsatellite instability (32), were directly purchased from ATCC (CRL-2638) and maintained for fewer than 5 passages. All *in vivo* experiments were performed on 6-8 week old BALB/c mice (The Jackson Laboratory, strain #:000651) after a minimum of 48 hours acclimatization, and all procedures were performed in accordance with guidelines and regulations set by the Wayne State University Institutional Animal Care and Use Committee. Inoculations were given subcutaneously on the right inguinal quadrant with 2×10^5^ CT26 cells suspended in sterile RPMI (Gibco, 72400047). Tumor growth was monitored by caliper measurement 3 times per week and tumor volume was calculated as (W×W×L)/2.

### Treatment with ICI

Mice were randomly distributed into treated and untreated groups. ICI was given by intraperitoneal injection with 200 µg anti PD-1 (clone RMP1-14, P362, Leinco Technologies) and 100 µg anti CTLA-4 (clone 9D9, C2855, Leinco Technologies) in 200 µL sterile saline.

### Radiochemistry and PET imaging

Radiochemistry of anti-mouse-IFNγ (clone AN18, Thermo Fisher Scientific) and IgG1 isotype control anti-horseradish peroxidase (clone HRPN, BE0088, BioXCell) was performed as described previously (22, 26). All antibodies were conjugated to p-SCN-Bn-Desferrioxamine (DFO) with a 1:5 mole ratio of mAb : DFO in saline at pH ~9 for 1hr at 37°C. Unbound DFO was removed via spin column centrifugation (MWCO: 30 kDa, GE Vivaspin 500). ^89^Zr (3D Imaging) was incubated with the mAb-DFO conjugates at pH ~ 7.2-7.4 at room temperature for 1hr. Unbound ^89^Zr was removed via spin column centrifugation (MWCO: 30 kDa, GE Vivaspin 500) using saline as eluent buffer. [^89^Zr]Zr-DFO-anti-IFNγ and [^89^Zr]Zr-DFO-IgG were each labeled at a specific activity of ~5 mCi/mg. Radiochemical yields of both constructs were >95% as determined via radio-instant thin layer chromatography (iTLC, Eckert & Ziegler). Tumor-bearing animals used for imaging were injected i.v. with radiolabeled antibodies (189 ± 31 µCi) in ~150 µL sterile saline. PET images were acquired 72 hrs post-injection on a Bruker Albira SI microPET/CT system. Images were decay corrected and analyzed in PMOD version 4.304. Volume of Interest (VOI) measurements within tumors were used to determine the uptake of the radiotracer, which is expressed as the maximum injected dose per mL (%ID/mL).

### Quantitative reverse transcribed PCR

Tumor tissue collected from mice was snap-frozen in liquid nitrogen and allowed to decay for >10 half-lives stored at -80°C. Tissue was then homogenized with a Tissue Tearor in TRIzol and RNA was extracted as described by the manufacturer (Thermo Fisher Scientific). cDNA was synthesized using a ProtoScript First Strand cDNA synthesis kit (New England Biolabs) using PolyDT primers. qRT-PCR was conducted with TaqMan probes (Thermo Fisher Scientific) for glyceraldehyde 3-phosphate dehydrogenase (GAPDH) (Mm99999915_g1), IFNγ (Mm01168134_m1), and IRF1 (Mm01288580_m1). 10 ng of cDNA/well was used and mRNA quantity was calculated as 2^-ΔCT^ relative to GAPDH. Transcripts that failed to amplify in all technical replicates were set to a CT value of 55. Transcripts in which only 1/3 of technical replicates amplified were removed.

### Data and statistical analysis

All imaging analysis was performed in PMOD (version 4.304). Statistical analysis utilized R (version 4.1.2) or GraphPad Prism (version 9.5.1). For all two-way ANOVA comparisons, a test of equal variance among groups was performed. If groups had unequal variance, a general least squares model was used to compare variable effects and to test whether variables interacted. A p-value < 0.05 is considered statistically significant. Statistical comparisons between groups were performed as stated in the figure legends.

## Results

### [^89^Zr]Zr-DFO-anti-IFNγ PET tracer shows specific uptake in tumors treated with ICI

**Figure 1:**
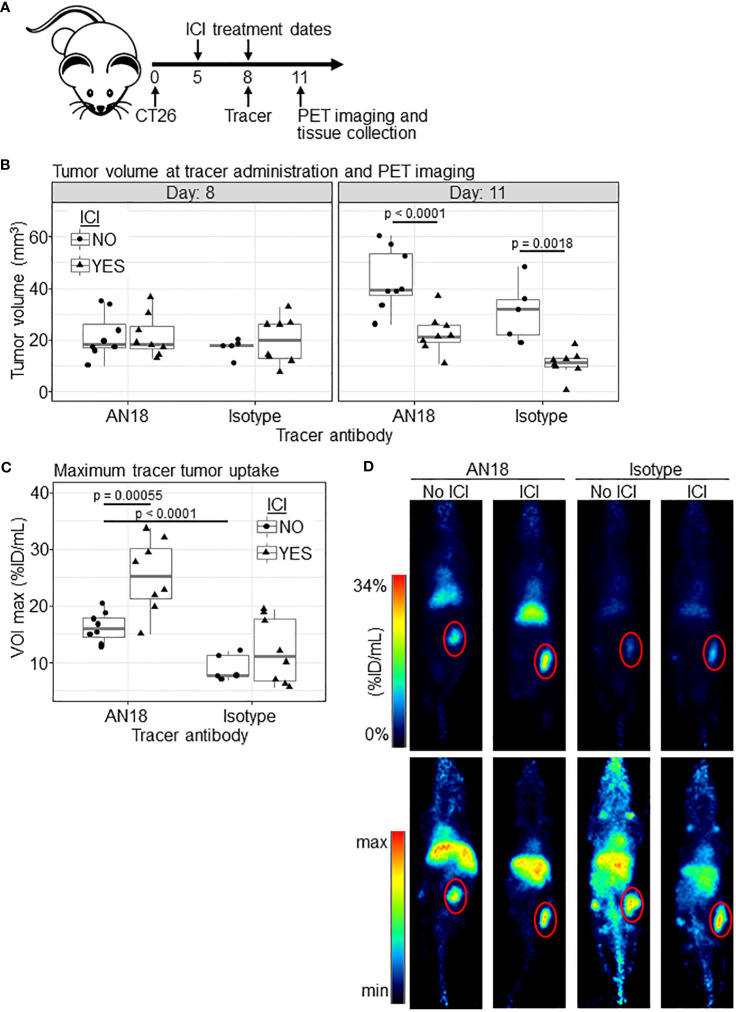
[^89^Zr]Zr-DFO-anti-IFNγ shows tumor-specific uptake in ICI-treated mice. **(A)** Schematic of the experiment. **(B)** CT26 tumor volumes for experimental groups (ICI NO, anti-IFNγ tracer n=8; ICI YES, anti-IFNγ tracer n=8; ICI NO, isotype control tracer n=5; ICI YES, isotype control tracer n=8) at both tracer injection (day 8, left panel) and PET imaging (day 11, right panel) timepoints. **(C)** Maximum PET tracer uptake VOI within the tumor in %ID/mL. **(D)** Representative PET images from animals closest to the mean %ID/mL for the group in **(C)**, with the tumor outlined in red. 2D coronal (top panel) and 3D maximum intensity projection (MIP) (bottom panel) are shown. Significance was determined via Tukey’s test after general least squares testing using an interaction model of tracer target and ICI.

To determine if an anti-IFNγ immunoPET tracer could identify intratumoral immune activity after ICI, we utilized BALB/c mice bearing subcutaneous CT26 colorectal cancer as a responsive model for combined ICI (anti-CTLA4 and anti-PD-1) therapy. Mice were inoculated with CT26 on day 0 and treated on days 5 and 8 with combined ICI. On day 8, mice were also given ~200 µCi [^89^Zr]Zr-DFO-anti-IFNγ or an IgG isotype control ([^89^Zr]Zr-DFO-anti-horseradish peroxidase) tracer, with PET imaging 72 hrs post-injection (**Figure 1A**) to allow for unbound tracer clearance and optimal imaging per our previous experience (26). At the time of tracer administration there was no significant difference in tumor volume between any of the experimental groups (**Figure 1B**, left panel); however, at the time of imaging on day 11 post-inoculation, tumor volumes were significantly lower in mice receiving ICI treatment (p<0.0001 for IFNγ, p=0.0018 for IgG, **Figure 1B**, right panel). Importantly, within the treatment groups there was no difference in tumor volume attributable to the IFNγ versus the isotype control tracer (**Supplementary Figure 1**).

Quantitation of tumor tracer uptake illustrated a significant increase in IFNγ tracer accumulation in tumors from ICI-treated (25.23 ± 6.48 %ID/mL) versus untreated mice (16.22 ± 2.68 %ID/mL, p<0.00055, **Figures 1C, D**). Comparatively, mice receiving the isotype control exhibit a marginal but insignificant difference in tumor tracer uptake between untreated (8.49 ± 1.98 %ID/mL) versus treated (12.06 ± 5.77 %ID/mL, p=0.394) animals (**Figures 1C, D**). Interestingly, there is also a significant difference in uptake between untreated (8.49 ± 1.98 %ID/mL) mice receiving the IgG isotype versus [^89^Zr]Zr-DFO-anti-IFNγ tracer (16.22 ± 2.68 %ID/mL, p<0.0001), which suggests baseline IFNγ expression may be present in untreated CT26 tumors. This observation is consistent with previous reports indicating CT26 is an immunogenic tumor model with moderate levels of IFNγ transcript in the TME (33–35). Consistent with our prior experience, non-specific antibody tracer accumulation is also evident in the liver, which is the primary organ for excretion. Biodistribution studies with this tracer have been performed previously (26).

### [^89^Zr]Zr-DFO-anti-IFNγ PET tracer uptake correlates with ICI treatment outcomes

**Figure 2:**
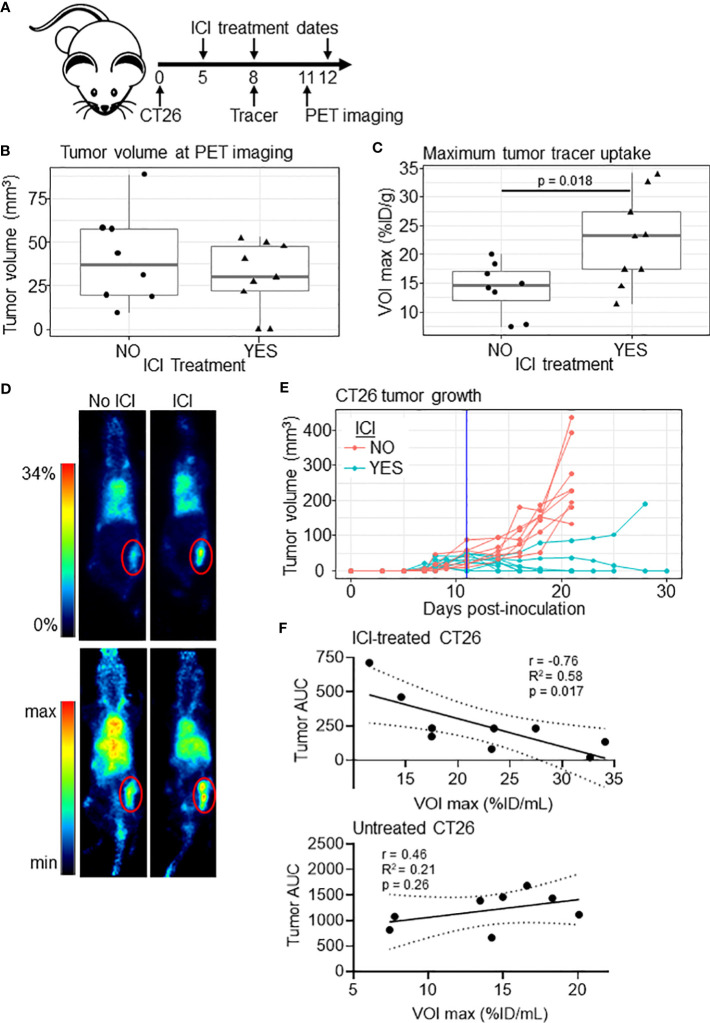
[^89^Zr]Zr-DFO-anti-IFNγ tumor uptake correlates to tumor burden in treated mice. **(A)** Schematic of the experiment. **(B)** Tumor volume of untreated (n=8) and ICI-treated (n=9) mice at the PET imaging timepoint (day 11). **(C)** Maximum tumor uptake of [^89^Zr]Zr-DFO-anti-IFNγ tracer VOI in %ID/mL for ICI-treated and untreated mice. Significance determined by Student’s T-test. **(D)** Representative PET images from animals closest to the mean %ID/mL for the group in **(C)**, with the tumor outlined in red. 2D coronal (top panel) and 3D MIP (bottom panel) are shown. **(E)** Tumor growth in mice bearing CT26 tumors with a vertical line indicating the PET imaging date. **(F)** Plot of tumor [^89^Zr]Zr-DFO-anti-IFNγ uptake relative to tumor burden measured by area under the curve (AUC) through day 21 for ICI-treated (top panel) and untreated (bottom panel) mice. The Pearson correlation coefficient (r), the coefficient of determination (R^2^), and p values are shown. Linear correlation is indicated with a solid black line, with a dashed line representing the 95% confidence interval.

We next tested whether tumor [^89^Zr]Zr-anti-IFNγ uptake would correlate with ICI outcomes. ICI-treated or control CT26-bearing BALB/c mice received ~200 µCi [^89^Zr]Zr-DFO-anti-IFNγ, PET imaging was conducted, and tumor volumes were monitored until they reached an experimental endpoint of ≥ 100 mm^3^ (**Figure 2A**). Unlike our initial experiment, at the time of imaging the tumor volume remained similar between treated and untreated groups (**Figure 2B**); however, we did observe a significant difference in tracer uptake (p = 0.018, **Figures 2C, D**). These results suggest that tracer accumulation is independent of tumor volume. Response to ICI is evident, as only 1/8 treated mice failed to eliminate the tumor (**Figure 2E**). To determine whether tumor tracer uptake correlated to the magnitude of ICI response, we calculated the area under the curve (AUC) of tumor volumes for each animal through day 21, at which point all untreated tumors had surpassed our experimental threshold and were euthanized. Within ICI-treated mice, we observed an inverse linear correlation of tumor burden AUC and tracer uptake within the tumor (r= -0.76, R^2 = ^0.58, p = 0.017, **Figure 2F**, upper panel). Comparatively, in untreated mice there was no significant association between these conditions (r=0.46, R^2 = ^0.21, p = 0.26, **Figure 2F**, lower panel), suggesting tracer uptake is indicative of ICI efficacy.

### The impact of the anti-IFNγ antibody tracer on downstream signaling

**Figure 3:**
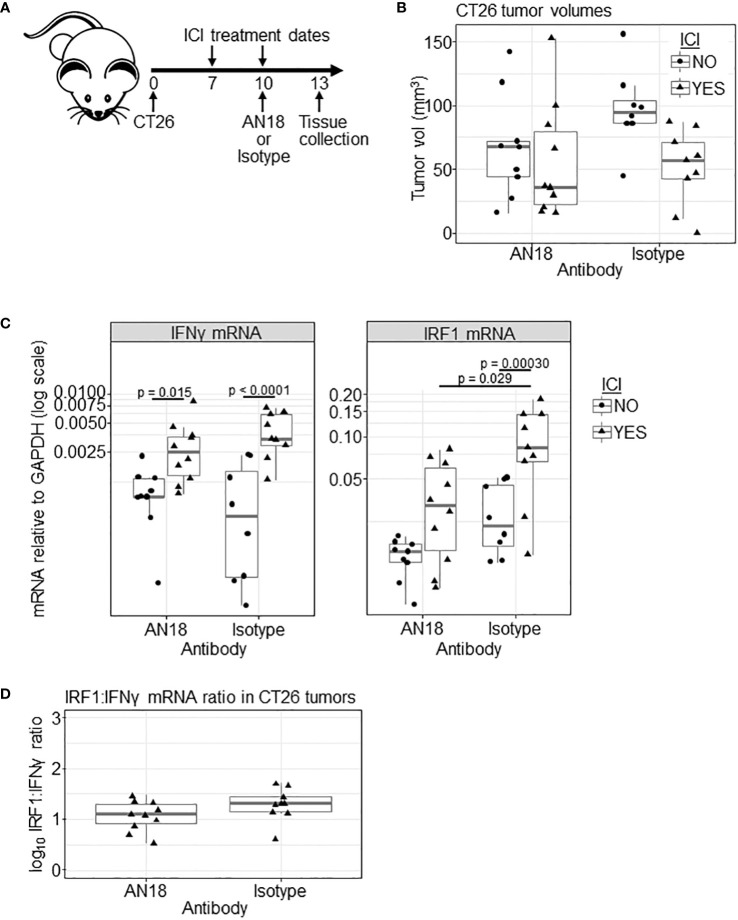
Effect of AN18 antibody on downstream signaling. **(A)** Schematic of the experiment. **(B)** CT26 tumor volume of experimental groups (ICI NO, AN18 antibody n=9; ICI YES, AN18 antibody n=10; ICI NO, isotype control antibody n=8; ICI YES, isotype control antibody n=9) on the day of tissue collection (day 13). **(C)** qRT-PCR for IFNγ (left panel) and IRF1 (right panel) mRNA from snap-frozen tumor sections. Transcript levels were normalized to GAPDH and are displayed on a log scale. **(D)** Log transformed ratio of IRF1 to IFNγ transcript abundance. Significance for **(B–D)** were determined via Tukey’s test after general least squares testing using an interaction model of tracer target and ICI.

Because most anti-IFNγ antibodies including clone AN18 are neutralizing, we next tested whether the tracer dose of antibody has a detrimental effect on IFNγ signaling. Unfortunately, ICI treatment on days 5 and 8 yielded inadequate residual tumor tissue within the treated cohort to extract quality RNA for downstream analysis of IFNγ expression and downstream signaling. To give the tumor more time to develop, we shifted the experimental timeline by two days, initiating ICI treatment on day 7, giving tracer antibody (without PET imaging) on day 10, and harvesting tissue on day 13 (**Figure 3A**). Upon tissue harvest, we observed no significant differences in tumor volumes between experimental groups (**Figure 3B**).

We performed qRT-PCR on mRNA extracted from snap-frozen tumor tissue, measuring transcripts for IFNγ, to indicate response to ICI, and interferon regulatory factor 1 (IRF1), to indicate whether downstream IFNγ signaling is disrupted. Upon IFNγ engagement with its receptor, the signal transducer and activator of transcription 1 (STAT1) protein is phosphorylated, translocates the nucleus, and initiates IRF1 mRNA expression (31). Compared to untreated controls, ICI treatment led to increased IFNγ mRNA for both AN18 and IgG control antibody-treated groups (p= 0.015 and p<0.0001, respectively, **Figure 3C**). IRF1 transcript, however, was only significantly increased in mice receiving isotype control antibody (p = 0.00030), which might suggest that downstream signaling was affected by AN18. In fact, IRF1 expression is higher in ICI-treated mice receiving the control antibody compared to ICI-treated mice receiving AN18 (p = 0.029). While IFNγ expression itself was not significantly different between these groups, there was a slight elevation of IFNγ transcript in mice receiving the control antibody. As an additional test to determine whether the AN18 antibody was impacting IFNγ signaling within the tumor, we compared the ratio of IRF1:IFNγ transcripts, since IRF1 expression is in part dependent on the presence of IFNγ (**Figure 3D**). There was no significant difference in the ratio of IRF1:IFNγ mRNA in ICI-treated mice receiving AN18 or IgG control tracer antibody (p = 0.24), suggesting downstream signaling was only marginally affected by AN18, if at all. Collectively these results warranted further evaluation of the impact that the anti-IFNγ tracer antibody has on ICI therapeutic efficacy.

### An imaging dose of anti-IFNγ antibody does not interfere with ICI response rates

**Figure 4:**
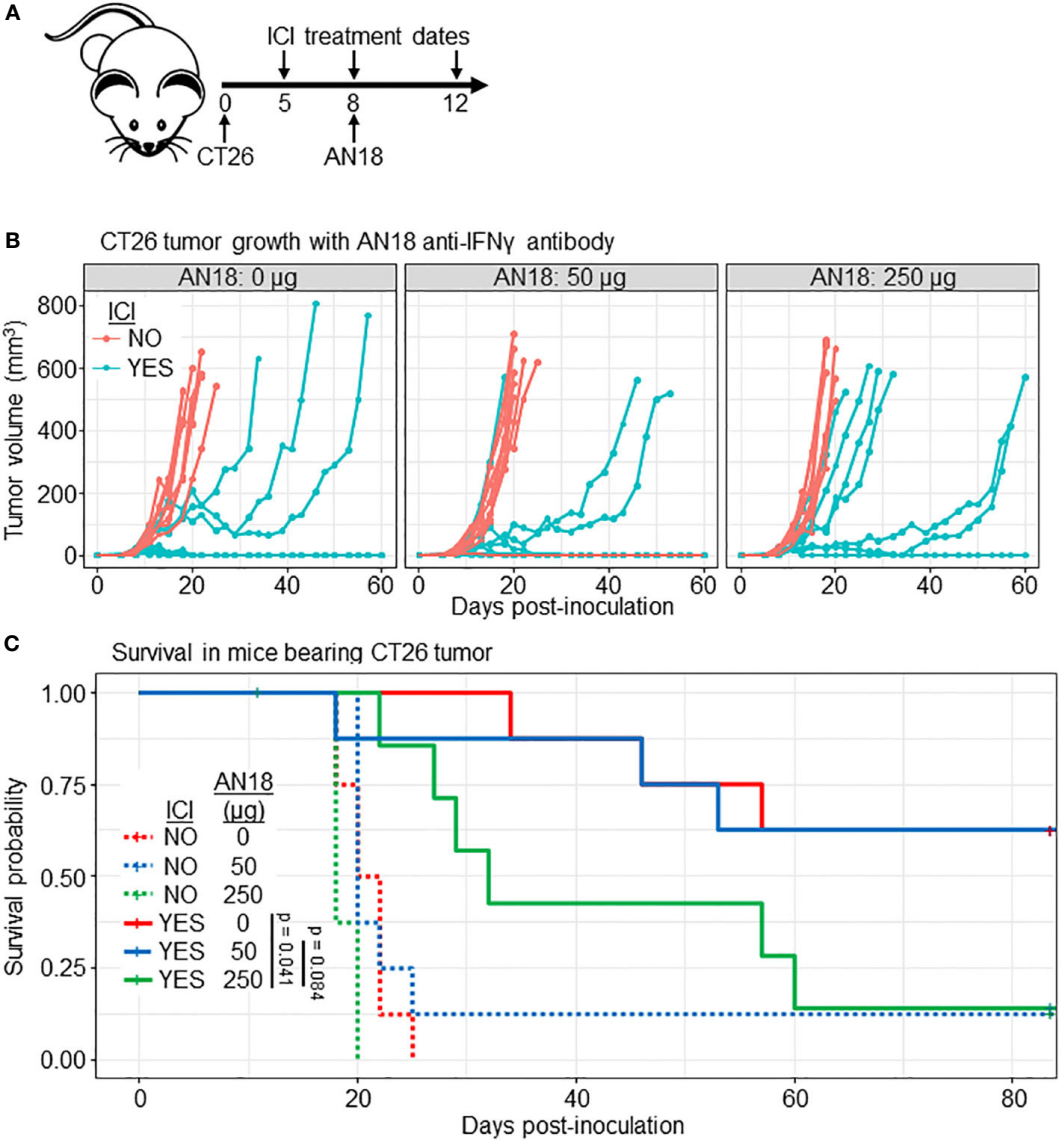
Imaging dose of AN18 antibody does not impact ICI efficacy. **(A)** Schematic of the experiment. **(B)** CT26 tumor volume of experimental groups (n=8 per group). **(C)** Kaplan-Meier survival plot from mice in **(B)** to depict survival differences among treated mice receiving different dose of anti-IFNγ antibody. Significance is determined by Log-rank (Mantel-Cox) test.

Due to our results from the qRT-PCR analysis, it was important to determine whether the use of an antibody-based IFNγ tracer could have a deleterious effect on ICI outcomes. Prior work has demonstrated that AN18 has a neutralizing effect in inflammatory conditions at a single 250 µg dose (36, 37). To investigate whether the tracer dose impacts ICI outcomes, we followed CT26 tumor growth in ICI-treated BALB/c mice receiving 0 µg, 50 µg (the imaging dose), or 250 µg (a neutralizing dose) of cold anti-IFNγ antibody with a treatment scheme matching our imaging experiments (**Figure 4A**). Mice receiving either 0 or 50 µg of anti-IFNγ had similar tumor growth curves with 5 of 8 mice eliminating tumor in each group (**Figures 4B, C**). ICI-treated mice receiving 250 µg AN18 tended to succumb to tumor, with only 1 of 8 mice achieving complete tumor regression. Survival analysis for ICI-treated mice across AN18 doses shows borderline significance between groups receiving 0 µg versus 250 µg (p = 0.041) and 50 µg versus 250 µg (p = 0.084) AN18. Importantly, there was no difference between survival of ICI-treated mice receiving 0 µg versus 50 µg AN18 (p = 0.95). This data suggests that while neutralization of IFNγ impedes ICI efficacy, the imaging dose of anti-IFNγ does not negatively impact therapeutic outcomes in this model.

## Discussion

Clinical monitoring of response to ICI remains difficult due in part to pseudoprogression and hyperprogression, both of which can be observed with traditional imaging during immunotherapy treatment (38, 39). This is partly resolved by the more recent iRECIST classification to determine progression, but better methods to predict and monitor response to ICI are still needed (40). The results of this study support the use of a PET tracer to the soluble cytokine IFNγ as a promising candidate for immunotherapy evaluation. We found that tumor-localized [^89^Zr]Zr-DFO-anti-IFNγ tracer uptake correlated with ICI treatment outcomes in CT26 tumor-bearing mice. Importantly, in most cases the elevated tracer uptake values preceded a treatment-induced reduction in tumor volumes, highlighting the potential for this tracer to identify intratumoral immune activity within the early stages of treatment.

We utilized an irrelevant IgG antibody to validate the specificity of the anti-IFNγ tracer, and while we observed that ICI treatment did not significantly increase control tracer uptake, we did see variability in the amount of tracer detected within tumors from this group (**Figure 1C**). This finding could be due to increased vascular permeability during an active immune response, which has been associated with expression of IFNγ and other cytokines (41). It has been shown that enhanced permeability and retention (EPR) can increase the concentration of macromolecules including IgG; however, there is little known regarding the effect of ICI on tumor vasculature and/or EPR, and it remains unclear whether these changes correlate with ICI response (42, 43). The variable uptake of the isotype control tracer after ICI may be an indicator of treatment-induced alterations within the TME that non-specifically contribute to antibody accumulation.

Our analysis of downstream signaling suggested that the tracer dose may have a physiologic effect, and thus we tested whether this was sufficient to hinder ICI outcomes. A cold anti-IFNγ tracer-dose of AN18 antibody did not have a negative effect on ICI therapeutic outcome, while a 5-fold higher, neutralizing dose dampened efficacy. IFNγ has a complex paradoxical relationship with both pro- and anti-tumor effects (28, 44–48). Recent studies have shown IFNγ has a profound effect on tumor signaling and can act as an inducer of ICI resistance (49–51). A mechanistic study in the B16 melanoma model demonstrated that IFNγ-driven resistance mechanisms may be due to signaling effects on tumor cells, and disruption of tumor-specific IFNγ signaling can rescue ICI sensitivity (49). It is possible that a sub-neutralizing dose of anti-IFNγ might dampen the pro-tumor effects of IFNγ signaling while preserving anti-tumor immunity.

Of note, anti-IFNγ tracer uptake was elevated compared to an isotype control tracer in untreated mice (**Figures 1C, D**). The CT26 tumor model is known to be immunogenic and responsive to immunotherapy (33–35), and our results suggest that anti-IFNγ PET may be able to detect localized pre-treatment IFNγ expression. This raises the question as to whether anti-IFNγ PET could be used to detect underlying immune activity, in turn identifying patients with historically non-responsive malignancies who may benefit from immunotherapy.

Previous studies have indicated that response to immunotherapy is dependent on characteristics of both the mouse strain and the specific tumor line (33, 35). We chose to utilize CT26-bearing BALB/c mice because of their responsiveness to ICI, but it will be important to expand upon our findings to include additional models. Our treatment scheme did not produce a 100% response rate, and thus we were able to correlate tracer uptake to tumor burden (**Figure 2F**). Importantly, tracer administration and/or imaging occurred prior to significant changes in volume between treated and untreated mice in most experiments, supporting their predictive value. However, future studies with less-responsive models would allow for a deeper investigation of whether the tracer can truly predict therapeutic outcomes in less ideal circumstances.

We found that tracer accumulation within the tumor correlated to tumor burden in our efficacy study (**Figure 2**); however, it is important to note that treatment was initiated 5 days after inoculation, and tumor burden was relatively low at the time of imaging (30.3 ± 19.8 mm^3^). Slower-growing tumor models, where treatment can be delayed until tumors are larger, may have more clinical relevance and provide more insight into the correlation of tumor burden to tumor tracer uptake. It will be important to determine whether IFNγ PET can identify intratumoral immune activity in patients treated with ICI, and whether tumor location or size influences imaging efficacy. It is anticipated that the liver would remain the primary route of excretion for a full-length antibody tracer (52), which may confound efforts to image primary hepatocellular carcinoma, or liver metastases derived from other tumor sites. Injection of excess unlabeled cold antibody immediately prior to tracer may ameliorate this effect (53); however in the case of IFNγ antibody, a 5X antibody dose suppressed ICI therapeutic efficacy (**Figures 4B, C**). Another possible alternative is the use of antibody fragments in place of full-length antibody. Liver accumulation may be reduced, however accumulation is often detected in other organs, including the kidneys (52). In addition to these considerations, an important first step to clinical translation will be the selection of a suitable anti-human IFNγ antibody clone, as AN18 does not cross-react with the human protein.

The immune landscape of cancer is complicated and biomarker discovery for immunotherapy has been full of promise, but definitive, dependable biomarkers have yet to be defined (54). Broad classifications of the cancer immune landscape cluster within, but also span across multiple cancer types (55). Consistent signatures of an ICI-responsive immune phenotype may also extend to historically less-responsive malignancies. Reliable, non-invasive biomarkers could help bolster clinical implementation of immunotherapy, and imaging modalities that capture immune activity within the tumor may serve an important role in monitoring and predicting therapeutic response. The findings of this study demonstrate the potential to meet this need with immunoPET tracers targeting IFNγ.

## Data availability statement

The original contributions presented in the study are included in the article/**Supplementary Material**. Further inquiries can be directed to the corresponding author.

## Ethics statement

The animal study was approved by Wayne State University Institutional Animal Care and Use Committee. The study was conducted in accordance with the local legislation and institutional requirements.

## Author contributions

JH: Formal Analysis, Investigation, Methodology, Validation, Writing – original draft, Writing – review & editing. NR: Methodology, Writing – review & editing. SB: Methodology, Writing – review & editing. MB: Methodology, Writing – review & editing. NV: Conceptualization, Writing – review & editing, Funding acquisition. HG: Conceptualization, Formal Analysis, Funding acquisition, Investigation, Methodology, Project administration, Supervision, Writing – original draft, Writing – review & editing.

## Supplementary material

Supplementary Figure 1 Plot of tumor volume grouped by treatment and tracer. Lines connect group means. Parallel lines indicate no interaction between treatment and tracer.
