# Supplementary material for: Interferon gamma immunoPET imaging to evaluate response to immune checkpoint inhibitors
Source: Front Oncol. 2023 Dec 7;13:1285117. doi: 10.3389/fonc.2023.1285117 (PMC10735274; doi:10.3389/fonc.2023.1285117)
Supplement: Supplementary Figure 1 — Plot of tumor volume grouped by treatment and tracer. Lines connect group means. Parallel lines indicate no interaction between treatment and tracer. [file DataSheet_1.pdf]

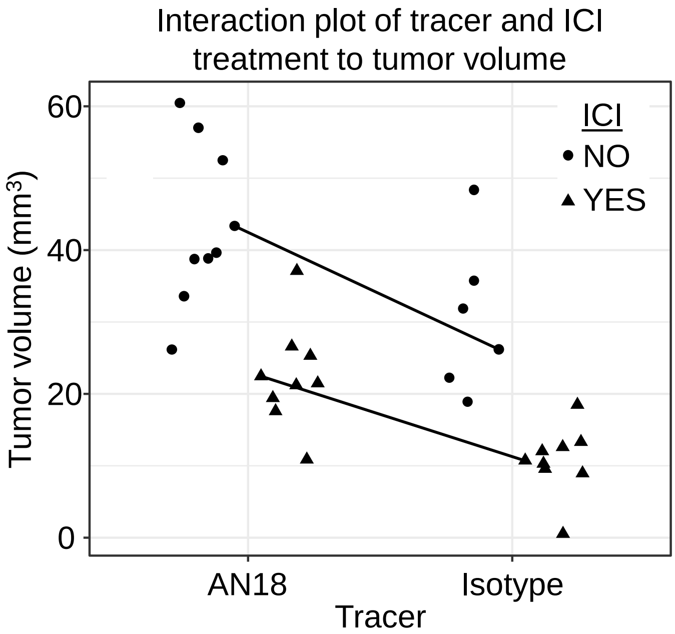

Supplemental Figure 1. Plot of tumor volume grouped by treatment and tracer. Lines connect group means. Parallel lines indicate no interaction between treatment and tracer.
